# Retraction Note: A very lightweight image super-resolution network

**DOI:** 10.1038/s41598-025-29851-0

**Published:** 2025-11-26

**Authors:** Haomou Bai, Xiao Liang

**Affiliations:** College of Computer and Communication, Lanzhou University of Technology, Gansu, 730050 China

Retraction of: *Scientific Reports* 10.1038/s41598-024-64724-y, published online 15 June 2024

The Editors have retracted this Article.

Following publication, the Editors have identified substantial overlap with previously published work, in particular^1,2^.

Neither of the Authors has responded to the correspondence from the Editors about the retraction.
